# Assessment of groundwater safety surrounding contaminated water storage sites using multivariate statistical analysis and Heckman selection model: a case study of Kazakhstan

**DOI:** 10.1007/s10653-020-00685-1

**Published:** 2020-08-08

**Authors:** Ivan Radelyuk, Kamshat Tussupova, Magnus Persson, Kulshat Zhapargazinova, Madeniyet Yelubay

**Affiliations:** 1 Department of Water Resources Engineering, Lund University, Box 118, 22100 Lund, Sweden; 2 Center for Middle Eastern Studies, Lund University, 22100 Lund, Sweden; 3 Department of Chemistry and Chemical Technology, Pavlodar State University, 140000 Pavlodar, Kazakhstan; 4 Kazakh National Agrarian University, 050010 Almaty, Kazakhstan

**Keywords:** Kazakhstan, Petrochemical industry, Water quality, Principal component analysis, Cluster analysis, Heckman selection model

## Abstract

Petrochemical enterprises in Kazakhstan discharge polluted wastewater into special recipients. Contaminants infiltrate through the soil into the groundwater, which potentially affects public health and environment safety. This paper presents the evaluation of a 7-year monitoring program from one of the factories and includes nineteen variables from nine wells during 2013–2019. Several multivariate statistical techniques were used to analyse the data: Pearson’s correlation matrix, principal component analysis and cluster analysis. The analysis made it possible to specify the contribution of each contaminant to the overall pollution and to identify the most polluted sites. The results also show that concentrations of pollutants in groundwater exceeded both the World Health Organization and Kazakhstani standards for drinking water. For example, average exceedance for total petroleum hydrocarbons was 4 times, for total dissolved solids—5 times, for chlorides—9 times, for sodium—6 times, and total hardness was more than 6 times. It is concluded that host geology and effluents from the petrochemical industrial cluster influence the groundwater quality. Heckman two-step regression analysis was applied to assess the bias of completed analysis for each pollutant, especially to determine a contribution of toxic pollutants into total contamination. The study confirms a high loading of anthropogenic contamination to groundwater from the petrochemical industry coupled with natural geochemical processes.

## Introduction

Safe drinking water is one of the sustainable development goals announced by the UN; however, in many countries, the goal remains far off. In 2015, the distribution of global groundwater use was estimated to 50% for drinking purpose and 43% for irrigation (UNESCO 2015). Historically, groundwater quality has been deteriorated by human activities, such as agricultural, industrial, and urbanization processes (WHO 2006). In Kazakhstan, groundwater withdrawal amounted to 1.078 km^3^ in 2016 (UN 2019). One crucial problem in the country is toxic wastewater from petrochemical factories (Radelyuk et al. 2019), a very important factor in Kazakhstani economy. The oil refinery industry is represented by three large factories, and their capacity is estimated to be 360 thousand barrels daily with an annual growth of 2.9% (BP 2018). Additionally, refineries are associated with petrochemical industry. Industrial clusters are established around core refineries. It leads to growth of production and increasing level of contamination. The problem is that the current methods of wastewater treatment in the petrochemical sector, as well as the conditions of the treatment units built during the Soviet era, do not assure a safe level of contaminants concentrations for the ecological systems. Thus, the existing discharge system has a significant negative impact on the environment and could potentially become a health issue for the population.

The groundwater is the main source for decentralized and centralized drinking water supply in rural areas in Kazakhstan, where more than half of the population live (Zhupankhan et al. 2018; Bekturganov et al. 2016). The perceived water quality has been assessed in several research and showed relative satisfaction (Tussupova et al. 2015, 2016). However, in situ water quality and potential risk for groundwater safety have not been covered within existing scientific literature. Simultaneously, petrochemical plants in Kazakhstan continue to discharge wastewater with high concentrations of different pollutants and these contaminants may reach the groundwater very easily. Despite of existing system of ecological monitoring, oil refinery cluster in Kazakhstan is ranked as one of the biggest sources of water contamination by United Nations Economic Commission for Europe (UNECE 2019). Recent studies showed that approximately 1.5% of total deaths in Kazakhstan caused by waterborne diseases related to water pollution, including industrial sources (Karatayev et al. 2017).

While contaminated sites occupy relatively small area, they belong to larger aquifers and potentially cause serious hazard (Maskooni et al. 2020). The contaminated sites are considered as a serious problem worldwide (Kovalick and Montgomery 2017). Moreover, the situation becomes worse if governments deny any environmental pollution or the contaminated sites are not investigated (Naseri Rad and Berndtsson 2019). Research-based approach can deal with the situations and helps do right decisions about remediation programs and protect population and environment from related risks (Naseri Rad et al. 2020). Thus, it is urgent to identify the main sources of groundwater pollution from petrochemical industry in Kazakhstan in order to eliminate the risks.

Multivariate statistical techniques have been widely used for assessment of surface and ground water quality (Shrestha and Kazama 2006; Naseh et al. 2018; Cloutier et al. 2008; Ghahremanzadeh et al. 2018; Noori et al. 2010; Patil et al. 2020). The natural transformations happen due to saltwater intrusion, lithological/geochemical processes, rainfall and snowmelt, eutrophication processes. The anthropogenic invasion due to urban development, industrial and agricultural activities, influence by rural settlements significantly contributes to groundwater pollution, and consequently, affects the water quality. Thus, multivariate statistical techniques are efficient tools identifying and separating the main probable sources of pollution in the context of land-use changes. Three techniques are particularly common: Pearson’s correlation, Principal Component Analysis and Cluster Analysis. Correlation matrix is used to determine potential interactions between different chemicals by pairwise variables comparison. PCA is used to identify statistically the most significant parameters, which are considered as major contributors to total contamination. Finally, CA combines similar groups of observations together. The techniques have been successfully applied, e.g., Egbueri (2019) divided his study area in Nigeria into insignificantly and highly polluted sites by using CA. Awomeso et al. (2020) investigated and identified possible sources of groundwater contamination such as leachate from septic tanks, nutrients from agricultural fields and chlorine pollution. The multiple natural and anthropogenic sources of surface and groundwater pollution have been presented by Omo-Irabor et al. (2008) in Nigeria. Impact on shallow groundwater in irrigated areas has been investigated by Trabelsi and Zouari (2019) in Tunisia. Shrestha and Kazama (2007) combined sites as less polluted, medium polluted and highly polluted, based on the similarities of water quality indicators in Japan. The same was for Kazi et al. (2009) who investigated the problem of water contamination by agriculture and industry in Pakistan. Liu et al. (2003) showed influence of processes of saltwater intrusion and arsenic pollution in Taiwan. Groundwater pollution sources apportionment in a land with high density of agriculture, industry and urbanization has been investigated in southwestern China (Li et al. 2019). Hence, the multivariate statistical techniques let researchers successfully investigate certain case studies.

The aim of this paper is to analyse and interpret a dataset obtained during a 7-year (2013–2019) monitoring program of the wastewater discharge systems in one of the Kazakhstani industrial clusters. This dataset includes concentrations of substances in groundwater from nine observed wells surrounding the wastewater recipient. Kazakhstani law (Kazakhstan 2015) requires that strict standards for groundwater quality surrounding recipients are followed. If the requirements are neglected, the responsible company should take actions to eliminate the risks for the environment and people. Matrix correlation, PCA, and CA multivariate techniques were applied to (1) determine main pollutants with elevated concentrations in groundwater, (2) assess the contribution of each contaminant to temporal variations in groundwater quality and identify their potential origin, and (3) group the contamination sites affecting water quality and their potential sources by relevant similarities. The results contribute to the description of the spatial–temporal changes in groundwater quality of the study area. Heckman selection model was used to avoid bias of the results and look at specific properties of each pollutant more carefully. Moreover, the study highlights the main sources of contamination at the different locations of the study area and is thus of interest for local key stakeholders, groundwater modelling researchers, and risk analysis managers.

## Materials and methods

### Study area

The industrial site of this study belongs to the special economic zone and is located in the north-eastern part of Kazakhstan. The region is located in a sharply continental zone, where mean monthly temperatures range from − 19.3 °C in January to + 21.5 °C in July, with an annual mean of 3.5 °C, absolute maximum of + 42 °C and absolute minimum of − 47 °C. Annual precipitation is around 303–352 mm, including 264 mm in liquid phase. The driest months are May, June, and July. Potential annual evaporation is around 957 mm (Heaven et al. 2007). Average relative humidity equals 82% and 45% for the coldest and the hottest period of the year, respectively. 70–85 days of the year is represented with the humidity 80% and more.

**Fig. 1:**
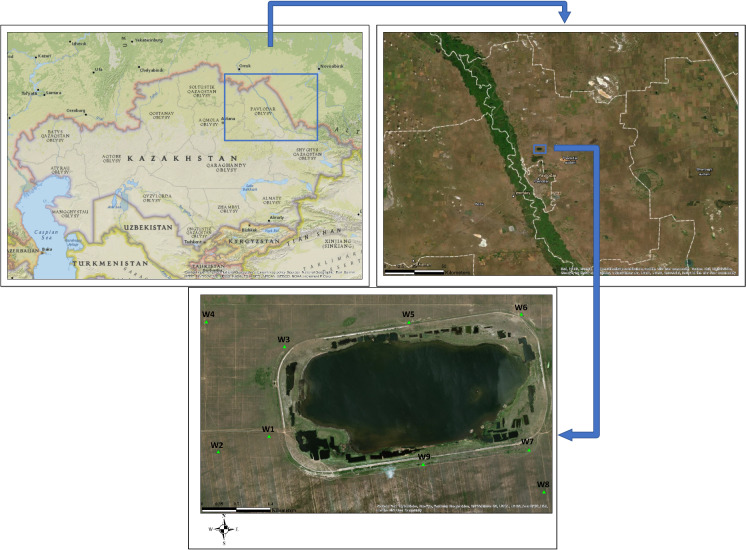
Study Area. Green triangles show location of wells sampled

The recipient pond (Fig. 1) is based on a natural bitter-salty pond for receiving and storing biologically treated wastewater from the nearby located petrochemical industry. According to Kazakhstani legislation (Kazakhstan 2012), this pond is not a source for drinking, domestic and irrigation water. The annual volume of received wastewater amounted to 1.63–2.21 million m^3^ for the period 2009–2019, instead of designed 4.12 million m^3^. The water volume and water surface for the same period are maintained within 3.6–6.7 million m^3^ and 2.45–3.73 km^2^, respectively, instead of the designed 23.5 million m^3^ and 5.23 km^2^, respectively. Observation wells are located out of barrier for groundwater quality monitoring and belong to permanent control from governmental bodies. The installation procedures followed appropriate installation technique in case of required installation materials and methods and planning of the location of the monitoring system (Houlihan and Lucia 1999). The depth of the wells varies between 10.1 and 24.6 m below ground level. The groundwater depth in the wells varied between 1.1 and 4.9 m.

**Fig. 2:**
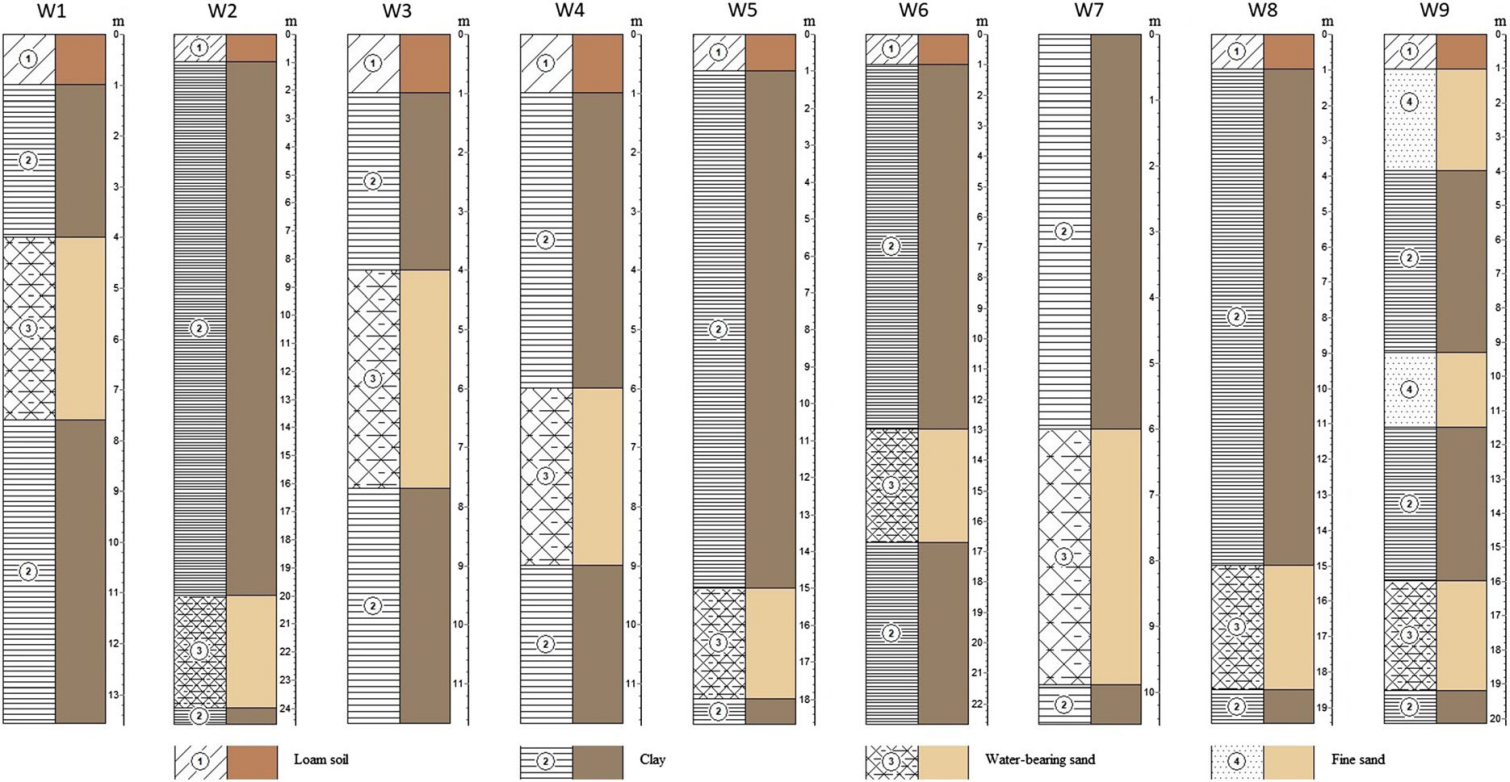
Geological profiles of the examined wells

**Fig. 3:**
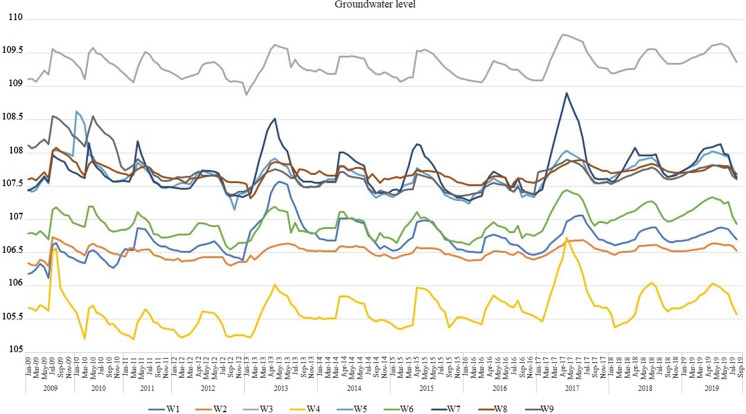
Seasonal fluctuations of groundwater level in the nine wells

**Fig. 4:**
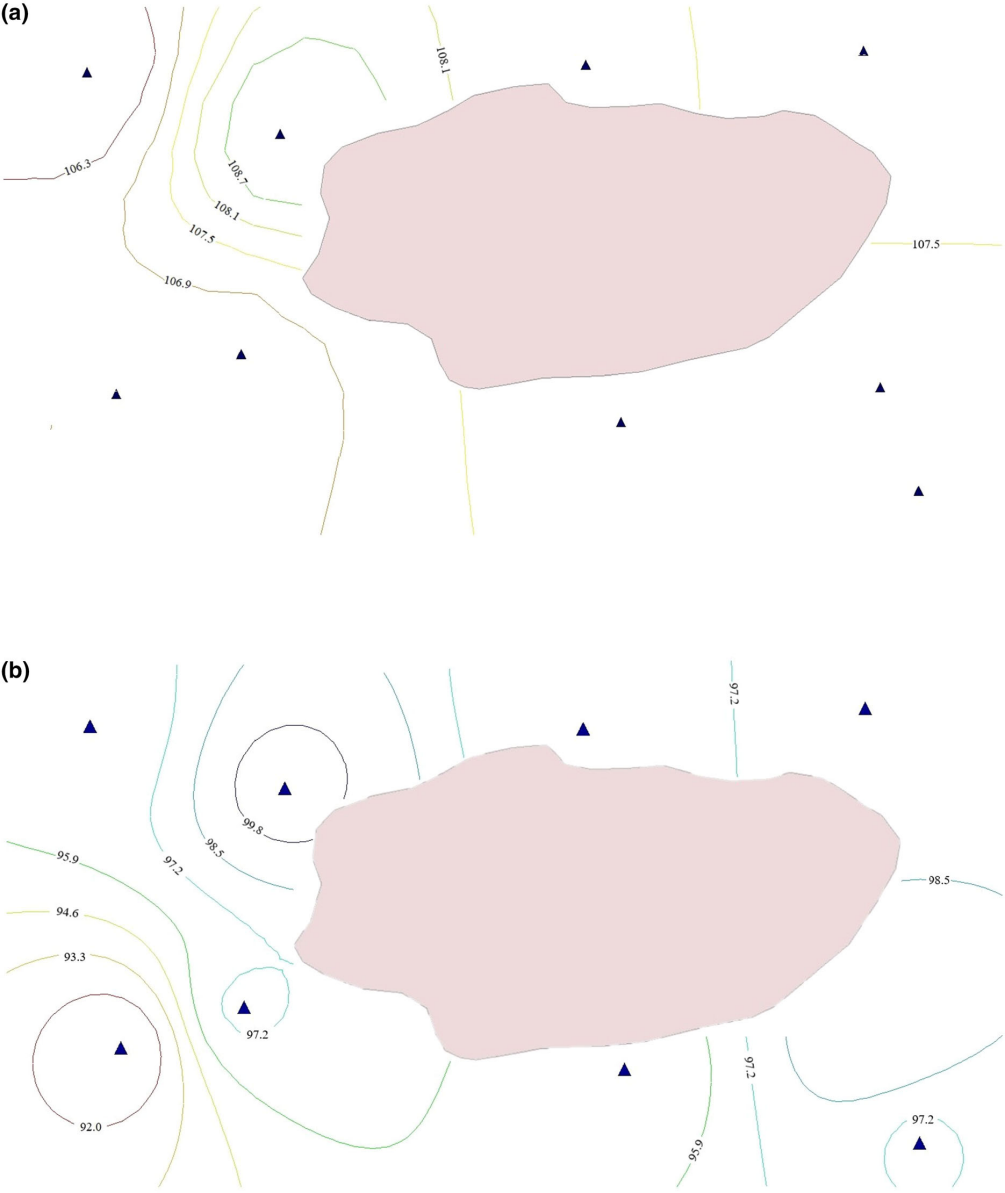
**a** Contour map of groundwater levels on the study area, **b** Spatial distribution of the bottom of the first aquifer

The hydrogeological conditions of the study area have been poorly investigated during soviet and post-soviet periods. The geological cross-section is represented by four geologic-genetic layers: contemporary sediments (land cover), upper-quarternary and contemporary aeolian–deluvial deposits (clayey sand) and upper-quarternary alluvial deposits (loam and/or fine to medium-grained sands). The geological profiles of the examined wells are presented in Fig. 2. Groundwater is represented by two aquifers: shallow unconfined and confined aquifers. The upper aquifer is composed of clay–sand and mixed size sands. The bottom of the aquifer lays on the depth 8.0–24.0 m below surface level. The aquifer is mainly recharged from water infiltration from the surface. The discharge is partly due to evapotranspiration and partly due to percolation to the underlying aquifer. Amplitude of seasonal fluctuation of groundwater table is about 0.7 m (Fig. 3). The figure shows that the GW level has peak values after the winter during the snowmelt season and after that reaches its minimal values during the summer. Interpolation using inverse distance method was used to establish GW flow direction and the bottom of the first aquifer. Figure 4 shows a contour map of the groundwater level and the elevation of the bottom of the unconfined aquifer. The second aquifer composed of medium-grained and small-grained sands. It is recharged from the head water and from the upper aquifer. The aquifer discharges to the nearest river, which is located 4 km west from the pond.

A total of 117 groundwater samples from the shallow aquifer were collected and analyzed between 2013 and 2019, from all observation wells. Sampling was made two times per year, in spring and autumn. The groundwater depth was measured regularly from March to November each year. The procedures of the sampling and measurements are controlled by Kazakhstani legislation from the sufficient international standards (Houlihan and Lucia 1999). Before sampling, the groundwater in the well was evacuated several times (usually, three times) by pumping. The pumping equipment was also flushed prior to sampling to avoid unwanted pollution. After establishing a static water level, the sampler was immersed to a depth below the water table by 0.5 m or less. Water samples were collected in 1-l dark glass bottles. The vessels were moved into a transportable fridge for immediate delivery and analysis to the licensed factory laboratory. Extra samples were collected for the analysis of metals with acidification by HNO_3_.

### Multivariate statistical techniques

Correlation analysis, principal components analysis (factor analysis), and hierarchical cluster analysis were applied to identify the multivariate relationships between different variables and samples in the study area. The dataset was normalized for elimination of the effect from differences in units (Eq. 1).

1 $$Z_{ij} = \frac{{\left( {x_{ij} - m_{i} } \right)}}{{{\text{SD}}}},$$

where *Z*_*ij*_ are normalized values from *x*_*ij*_, *i* is represented variables, *j* is the sample number, *m*_*i*_ is the mean value and SD is the standard deviation of the sample.

The relation between each pair of variables was measured by Pearson’s correlation coefficient to determine the geochemical associations among different variables. Correlation coefficients greater than 0.5 were considered significant. PCA recognizes the most significant parameters from a big dataset of inter-correlated parameters and created independent variables (Eq. 2).

2 $$z_{ij} = a_{i1} x_{1j} + a_{i2} x_{2j} + \cdots + a_{im}x_{mj} ,$$

where *z* is the component score, *a* is the component loading, *x* is the measured value of variable, *i* is the component number, *j* is the sample number and *m* is the total number of variables. Factor analysis (FA) is a similar technique as PCA. However, PC is presented as a linear combination of parameters. FA follows PCA and takes into account unobservable, hypothetical, and latent variables. They are included in equation with the special residual term (Eq. 3).

3 $$z_{ij} = a_{f1} f_{1j} + a_{f2} f_{2j} + \cdots + a_{fm} x_{mj} + e_{fi} ,$$

where *z* is the measured variable, *a* is the factor loading, *f* is the factor score, *e* is the residual term according to errors or other source of variation, *i* is the sample number and *m* is the total number of factors.

Cluster analysis was used to assemble similar groups of observed wells due to similarities between their variables. Hierarchical agglomerative CA provided Ward’s linkage distance, reported as *D*_link_/*D*_max_, which represents the quotient between the linkage distances for each case divided by maximal linkage distance. Produced dendrogram lets to analyse similarities easily. Ward’s linkage, the Euclidean distance as similarity measurements, and Q-mode are usually used for cluster analysis for assessment of groundwater quality (Egbueri 2019; Cloutier et al. 2008; Kazi et al. 2009; Awomeso et al. 2020; Trabelsi and Zouari 2019; Amanah et al. 2019; Bouteraa et al. 2019).

### Heckman selection analysis

Heckman selection analysis, to the authors’ knowledge, has never been applied to assess the environmental characteristics. This type of analysis was adapted from the original work of Heckman in the economical science (Heckman 1979) and from the application this type of this method in other fields, for example in the assessment of energy production (Sun et al. 2014), urban transportation research (Kaplan et al. 2016) and estimating crash rate (Xu et al. 2017). The method in this study is used to assess unobservable variables, that potentially impact on the total contamination rate. Gadgil investigated the list of chemicals in the WHO guidelines (Gadgil 1998) and concluded that certain chemicals have no strong requirements for their concentrations in drinking water, as the exposure of exceeded concentrations for human health is not significant. The idea of this assessment is not just looking at several contaminants and their concentrations, but also to consider and evaluate other important factors such as location of the sampled value, percent of exceeding of the certain contaminant and individual characteristics of the contaminant. Selected variables were divided into two categories. First: chemicals seriously affecting health (rated as sanitary toxic due to Kazakhstani standard (Kazakhstan 2015)); second: other hazardous materials (rated as non-toxic). It is aimed to compare potential effect of toxic and non-toxic contaminants. We focused, on the one hand, on several pollutants with elevated concentrations, such as chlorides or sulfates, which are not rated as significant impact on health, but can be dangerous for other cases, for instance, for corrosion of pipes, or for irrigation properties of soil; on the other hand, on the contaminants, rated as dangerous for the health, or toxic (for example, hardness or petroleum hydrocarbons).

This model includes two-step equation, which is assumed as an advanced regression model equation:

4 $$Y_{i} = \beta_{1} S_{i} + \beta_{2} X_{i} + u_{i} ,$$

where *Y*_*i*_ is considered as total contamination, *S*_*i*_ represents the concentration of chemicals, and *X*_*i*_ shows several contaminants as a set of control variables. The effect of the exceeded concentrations on the total contamination is given by the parameter *β*_*1*_. Parameter *i* represents each individual observation.

Equation (4) does not consider other potentially important independent variables which can affect for final result. For example, it could be locations of the wells or individual characteristics of different contaminants such as their toxicity and exposure level in case of influence of chemicals for people’s health. There could be a different input of high exceeding of non-toxic contaminant and low exceeding of toxic contaminant. Second one would be much more dangerous for health. Thus, more attention should be paid to the level of toxicity. Specific description of this equation can be written as:

5 $$\begin{aligned} Y_{i}^{*} & = \beta_{1} S_{i} + \beta_{2} X_{i} + u_{i} \\ D_{i} & = 1(\gamma_{1} S_{i} + \gamma_{2} Z_{i} + \nu_{i} > 0),\;{\text{and}} \\ Y_{i} & = Y_{i}^{*} D_{i} , \\ \end{aligned}$$

where (*Y*_*i*_*, D*_*i*_*, S*_*i*_*, X*_*i*_*, Z*_*i*_) are observed random variables and 1(.) is an indicator function. The first equation represents the total contamination of all contaminants. The second equation is the selection equation, where *D*_*i*_ is added as a dummy variable indicating whether value *i* represents a measurement of toxic/non-toxic pollutant. A set of variables *Z*_*i*_ includes additional parameter such as a well value *i*. Set of control variables *Z*_*i*_ must include at least one variable which is not included in *X*_*i*_ (Sartori 2003).

All mathematical and statistical computations were performed using Microsoft Office Excel 2016, IBM SPSS Statistics 26 software and STATA 15.0 (StataCorp LP).

## Results and discussion

### Groundwater quality parameters

Table 1 presents the results of measurements of groundwater quality from the wells surrounding the recipient pond. Kazakhstani and WHO standards for drinking water were used for assessing all parameters. The concentrations of several parameters in wastewater, which are discharged into the recipient pond, are also presented in the table. Those characteristics came from the previous publication of the authors (Radelyuk et al. 2019).

**Table 1:** Water quality parameters for groundwater samples from the observed wells. All units are in mg/L, excluding pH (pH unit) and total hardness (mmol/L)

Parameters	WHO limits* (WHO 2017)	KZ limits (Kazakhstan 2015)		W1	W2	W3	W4	W5	W6	W7	W8	W9	Effluents
pH	6.5–8.5	6–9	Range Mean SD	7.2–8.8 8.3 0.4	7.5–9.0 8.2 0.5	8.0–9.1 8.6 0.4	7.9–9.3 8.8 0.4	8.5–9.5 8.8 0.3	8.7–9.5 9.0 0.2	6.9–9.1 8.6 0.6	8.3–9.1 8.7 0.2	6.9–8.7 8.1 0.7	
TPH	0.1	0.1	Range Mean SD	0.16–1.04 0.44 0.25	0.11–1.40 0.41 0.38	0.09–0.60 0.29 0.14	0.11–0.84 0.39 0.24	0.08–1.20 0.40 0.33	0.14–0.67 0.41 0.17	0.23–0.78 0.51 0.19	0.11–0.99 0.45 0.28	0.26–0.84 0.52 0.18	0.68–2.15 1.23 0.40
TDS	1000	1000	Range Mean SD	846–1582 1156 185	4728–7727 6307 952	1346–2224 1779 299	643–2919 2072 561	899–1450 1218 172	683–1402 1046 203	1244–1933 1485 205	1157–1927 1470 298	28,202–36,392 31,848 2922	4–9 7 1
Cl^−^	250	350	Range Mean SD	98–211 158 32	2450–4410 3285 577	150–520 256 90	56–715 425 165	160–4410 549 1161	110–200 172 24	150–370 256 62	180–798 322 150	10,000–24,757 14,797 3372	50–135 83 26
SO_4_^2−^	250	500	Range Mean SD	94–210 164 33	544–1300 974 190	252–520 379 85	127–1100 670 231	89–284 208 46	150–849 298 179	214–296 260 22	305–443 351 39	4126–9400 7040 2086	238–589 449 91
Phenols**	–	0.25/0.001	Range Mean SD	0.00–0.04 0.01 0.01	0.00–0.01 0.00 0.00	0.00–0.06 0.01 0.01	0.00–0.06 0.01 0.02	0.00–0.06 0.01 0.02	0.00–0.06 0.01 0.02	0.00–0.05 0.01 0.01	0.00–0.05 0.01 0.01	0.00–0.12 0.02 0.04	0.01–0.03 0.02 0.01
NH_4_^+^	1.5	2	Range Mean SD	0.0–1.0 0.4 0.3	0.0–27.1 2.8 7.4	0.0–0.8 0.3 0.3	0.0–0.6 0.3 0.2	0.0–8.6 0.9 2.3	0.0–5.6 0.7 1.5	0.0–8.8 1.0 2.4	0.0–10.9 1.2 2.9	0.0–25.8 5.6 7.9	38.6–54.3 49.3 6.0
NO_2_^−^	3	3	Range Mean SD	0.0–0.6 0.1 0.2	0.0–2.0 0.2 0.6	0.0–1.1 0.2 0.3	0.0–0.7 0.1 0.2	0.0–0.4 0.1 0.1	0.0–0.4 0.1 0.1	0.0–0.7 0.2 0.2	0.0–0.9 0.1 0.3	0.0–14.5 1.5 4.2	0.1–4.4 0.1 1.3
NO_3_^−^	50	45	Range Mean SD	0.0–7.5 2.3 2.9	0.1–4.3 1.7 1.2	0.1–3.0 0.9 0.9	0.0–5.0 1.5 1.6	0.0–5.3 1.8 1.9	0.0–4.9 0.9 1.4	0.0–4.3 1.3 1.6	0.0–4.1 1.5 1.6	0.0–21.0 7.7 8.1	1.8–16.4 12.5 4.0
PO_4_^3−^	–	3.5	Range Mean SD	0.00–0.75 0.11 0.21	0.00–0.26 0.04 0.07	0.00–0.20 0.05 0.06	0.00–1.00 0.10 0.27	0.00–0.68 0.10 0.18	0.00–0.41 0.06 0.11	0.00–0.21 0.06 0.07	0.00–0.76 0.10 0.21	0.00–0.08 0.03 0.02	
CO_3_^2−^	–	–	Range Mean SD	0–48 15 15	0–15 6 5	0–87 30 28	5–59 32 17	6–36 20 8	13–84 31 18	0–137 39 37	0–73 26 19	0–45 11 13	
HCO_3_^−^	384***	–	Range Mean SD	189–494 375 92	14–391 119 126	329–709 537 100	43–514 326 110	262–512 343 74	201–346 277 42	9–578 374 176	210–329 264 37	66–464 201 138	
TH	–	7.0	Range Mean SD	3.7–12.5 7.6 2.0	6.2–67.9 54.0 15.6	3.2–9.8 6.8 1.7	3.0–12.0 7.6 2.6	2.1–5.0 3.9 0.9	2.0–4.8 3.8 0.9	2.4–9.3 6.5 1.7	2.8–15.1 6.5 3.5	219.0–390.0 272.0 55.0	
Ca^2+^	100	–	Range Mean SD	14–116 39 30	15–625 135 154	6–73 21 17	8–138 29 34	6–44 14 10	5–43 13 10	3–137 27 36	9–72 20 16	97–2844 497 714	
Mg^2+^	50	–	Range Mean SD	17–92 63 20	65–680 499 196	5–330 88 78	31–130 78 32	14–88 42 18	12–61 39 13	25–110 66 25	21–200 66 44	382–4422 2697 940	
K^+^	12	–	Range Mean SD	0.1–3.0 1.5 1.0	0.0–6.4 1.7 2.1	0.0–5.0 1.4 1.6	0.0–6.0 1.6 2.3	0.0–5.0 1.5 1.7	0.0–4.0 1.4 1.2	0.0–6.0 1.8 2.2	0.0–4.0 1.6 1.4	0.01–42.0 14.3 16.2	
Na^+^	200	200	Range Mean SD	140–230 190 27	605–1414 1136 272	390–685 480 85	66–775 545 181	220–540 348 94	200–500 296 77	290–560 390 79	220–680 412 135	5100–9200 7093 1377	
Surfactants	–	0.5	Range Mean SD	0.1–0.7 0.4 0.2	0.2–0.6 0.3 0.1	0.1–0.6 0.4 0.2	0.0–0.4 0.2 0.1	0.0–0.4 0.2 0.1	0.0–0.8 0.2 0.2	0.3–0.9 0.6 0.2	0.0–0.4 0.1 0.1	0.3–1.4 1.0 0.3	0.2–0.5 0.3 0.1
CO_2_	–	–	Range Mean SD	0–37 5 11	0–22 5 7	0–29 4 9	0–15 1 4	0 0 0	0 0 0	0–23 2 6	0–2 0 1	0–32 7 12	

– non-described

*WHO does not cover all chemical contaminants in the guidelines, but only those, which pose a risk in a high level (Gadgil 1998)

**EPA, EU and WHO present a range of phenol-derivatives according their toxicity rate. Kazakhstani standard assumes “phenols” as phenolic compounds, which evaporate under high temperature (Angelino and Gennaro 1997)

***From WHO Guidelines for drinking water quality (1984)

**Fig. 5:**
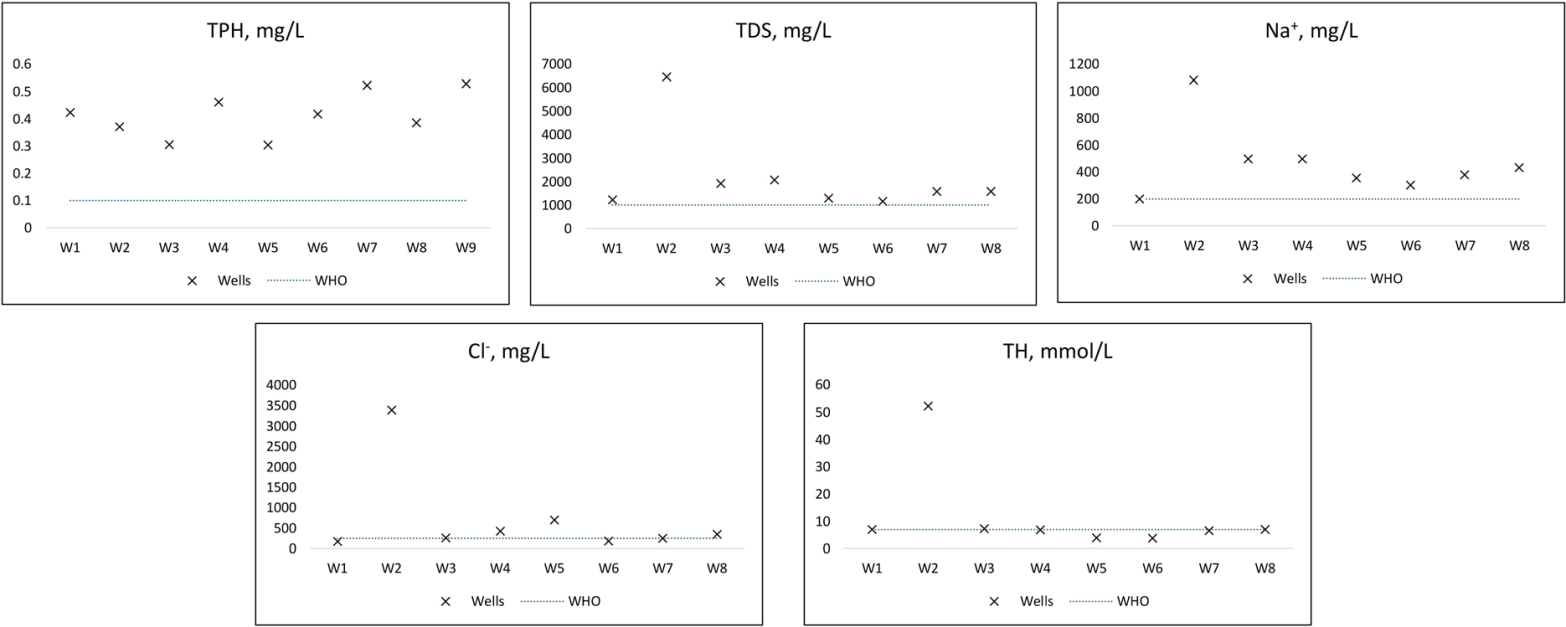
Concentrations of some chemicals in the groundwater wells compared to WHO limits

As shown, all wells had exceeding concentrations of total petroleum hydrocarbons (see also Fig. 5). When the permissible concentration of TPH is 0.1 mg/L, the concentrations of TPH varied between 0.08 and 1.20 mg/L with mean value 0.42 mg/L, which exceeded the norm 4 times. Although low concentrations of TPH in water might be considered harmless, researchers found that long-term exposure to TPH causes carcinogenic diseases (Pinedo et al. 2013; Wake 2005). Table 1 also shows that dangerous concentrations of phenols were identified in all nine wells. This pollutant had been evaluated as very toxic and was included in the list of priority pollutants by Environmental protection agency (EPA 2012). The number of disorders has been discovered by acute exposure of phenol: muscular convulsions, hypothermia, muscle weakness and tremor, collapse, coma, etc. (Nair et al. 2008). There is a limitation in the assessment of the presence of phenol in our case study. However, the limit of the concentration of simple phenol (phenol index) is 0.25 mg/L according Kazakhstani standard. The same value is established in the standard of the factory for the observed wells (Radelyuk et al. 2019). At the same time, protocols of GW quality measurements name this parameter “volatile phenols”. This type of phenolic compound is considered to be limited 0.001 mg/L. Thus, there is unclear situation of what limit should be used. Measured TDS values exceeded the KZ and WHO maximum permissible levels of 1000 mg/L in most cases on average five times (Fig. 5). Further, the total hardness in the groundwater samples ranged between 2 and 390 mmol/L with mean exceeding the standard six times (Fig. 5). According the Todd classification, almost all samples might be categorized as very hard water. Hard water may cause cerebrovascular and cardiovascular diseases (Stambuk-Giljanovic and Stambuk 2005). The chloride ion presence were between 56 and 24,757 mg/L, with most samples elevated WHO’s 250 mg/L recommended limit (exceeding 9 times on average) (Fig. 5). There are possible health-related concerns regarding Na^+^ content in the groundwater because the mean elevated concentrations in the wells were six times over the permissible KZ limits and WHO indirect recommendation (Fig. 5). Consumption of high amount of sodium has been correlated with cardiovascular disease, such hypertension and stroke (Lucas et al. 2011). Finally, individual exceedings of surfactants were identified. Such high level of surfactants is related to several potential problems. The presence of some surfactants in connection with other contaminants may decrease the biodegradation rate of contaminant or stops the process at all. In other cases, the presence of the surfactants enhances the biodegradation rate. The desirable result is not clear without knowing the role of the surfactant participating the biodegradation process in a given remediation plan (West and Harwell 1992). Moreover, special focus should be paid to Well 9 which had extremely high values. For example, TDS had a value 37 times above the limit, chloride 99 times higher than limit, sulphate exceeded the limit 38 times, total hardness with associated cations by 56 times as well as highly elevated concentrations of ammonia, nitrites, nitrates, potassium, sodium and surfactants (Table 1). This is the reason why Fig. 5 does not include Well 9 presenting the concentrations of some chemicals comparatively with WHO recommendations.

The water containing such levels of those substances would normally be rejected by consumers. Additional epidemiological research should be provided in municipalities nearby the area of pond to assess potential connections between the high concentrations of some parameters, such as TPH, phenols, Na^+^, Cl^−^, SO_4_^2−^, TDS and TH and cardiovascular and oncological diseases in the region.

**Fig. 6:**
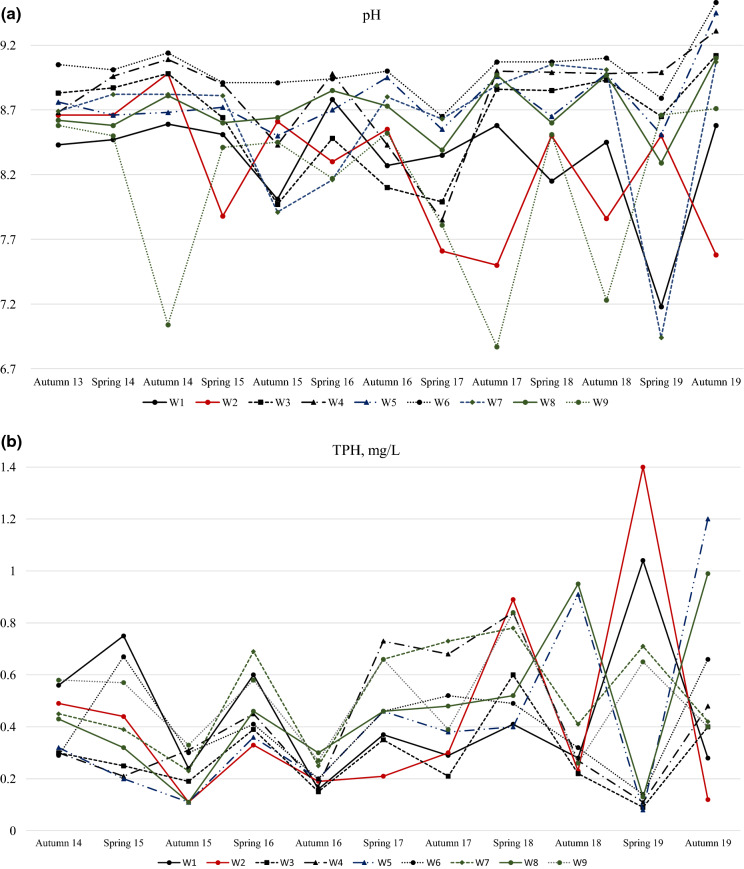

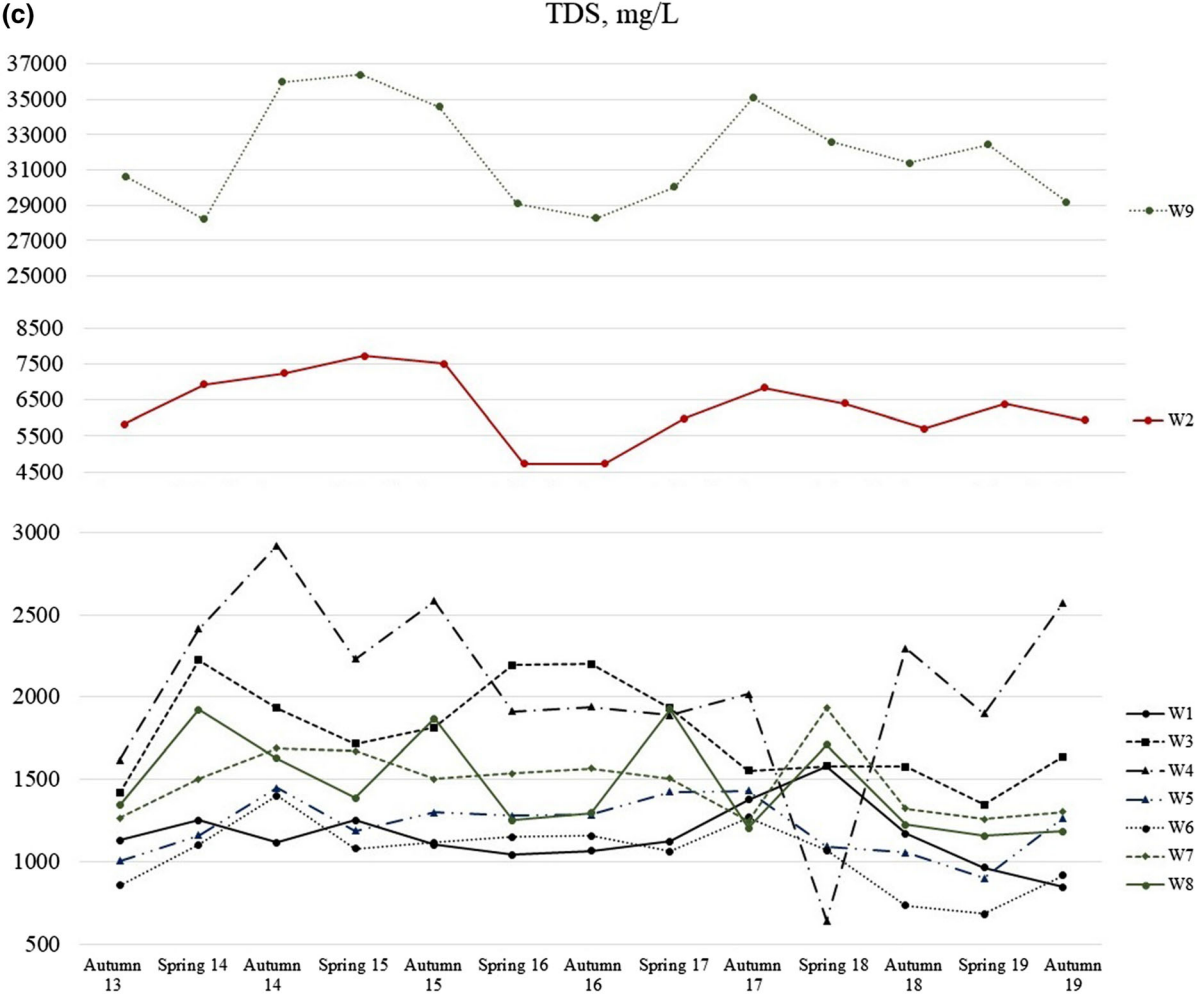
Temporal variation of **a** pH, **b** TPH and **c** TDS

Figure 6 shows temporal distribution of some chemicals. The pH values (Fig. 6a) normally were highest during the spring, while the value for W9 differs significantly and instead shows the lowest values during the same period. It could be explained by influence of recharge of snowmelt and geological characteristics of the area. The same situation can be applied for TPH. All wells show the highest concentrations of TPH during the spring (Fig. 6b). Moreover, the graphs mainly tend to raise their fluctuations and display an increasing trend. It potentially says that the pollution problem is growing in the area. Figure 6c represents the fluctuation of TDS in the groundwater. There are relatively flexible lines without significantly extremal changes.

**Fig. 7:**
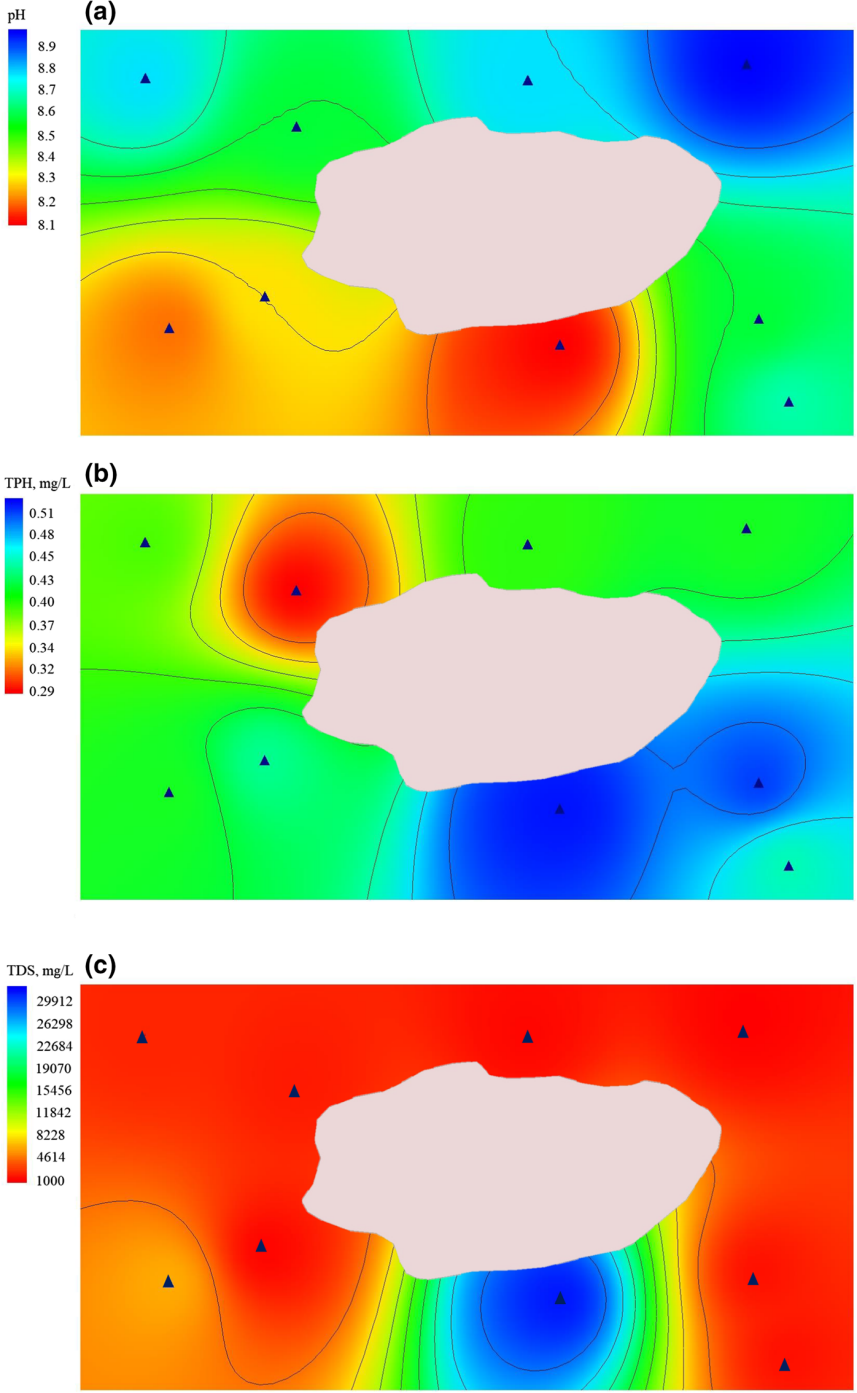
Spatial distribution patterns of **a** pH, **b** TPH and **c** TDS

**Fig. 8:**
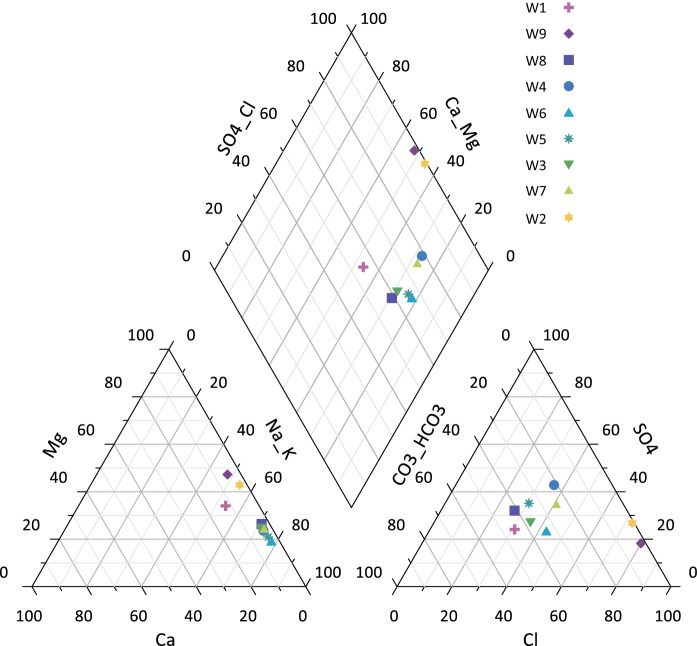
Piper diagram for identification of water type of the study area

Spatial distribution of the chemicals is presented in Fig. 7. pH values (Fig. 7a) are more than 7 for all wells, defining groundwater alkaline. According to Hem (1970) dissociation of carbonate and carbonate salts is a dominant process in nature, which leads to pH above 7. The maximal value of pH is found in well 6, and minimal value belongs to the wells 2 and 9. Piper diagram is widely used to show the dominant hydrogeochemical faces (Piper 1944). The Piper plot (Fig. 8) verifies the direct relationships between the hydrochemical regime of groundwater in the area and the pH value. Total petroleum hydrocarbons have a maximal value in the well 9 and minimal in the well 3 (Fig. 7b). There are plotted only TDS, instead of TH, Ca^2+^, Mg^2+^, Na^+^, Cl^−^ and SO_4_^2−^, on the figure, because they are parts of the TDS and distributed in the same manner (Fig. 7c). Thus, we can consider from the hydrogeological characteristics of this site (Fig. 4) and spatial distribution of pH and pollutants (Fig. 7) that groundwater flow has a slope toward western direction from the pond.

### Principal component analysis

The correlation matrix (Table 3) was employed for all 117 measurements for determining the loads of the principal components (PCs) shown in Table 2. The first six PCs were selected for the following reasons as variables of dimensionality reduction: the six PCs together gave a cumulative contribution of 78.34%, which is typically regarded as being sufficiently high; the eigenvalues of these PCs are all greater than 1.0 and, according to the Kaiser criterion these PCs must be chosen (Table 2) (Kaiser 1958). The factors can be conditionally divided into two groups. First group accounts to 52.34% of the total variance and is represented by Factors 1 and 2. Usually, the parameters, belonging to those factors, characterize natural conditions of the groundwater. Factors 3–6 contribute to 26% of the total variance and can be categorized, as anthropogenically appeared factors. The detailed interpretation of each Factor is explained below.

**Table 2:** Factor loadings (Varimax normalized)

	Natural		Anthropogenic			
Variable	Factor (1)	Factor (2)	Factor (3)	Factor (4)	Factor (5)	Factor (6)
pH	− 0.233	**− 0.900**	− 0.045	0.042	0.023	0.014
TPH	0.102	− 0.034	− 0.267	**0.746**	− 0.024	− 0.201
TDS	**0.924**	0.205	0.171	0.158	− 0.002	− 0.058
Cl^−^	**0.888**	0.251	0.203	0.146	− 0.086	− 0.058
SO_4_^2−^	**0.927**	0.086	0.203	0.063	0.041	− 0.046
NH_4_^+^	0.289	− 0.045	0.198	**0.783**	− 0.051	0.162
NO_2_^−^	0.254	− 0.059	**0.797**	− 0.075	− 0.173	− 0.004
NO_3_^−^	0.514	0.012	0.500	− 0.031	0.363	0.121
PO_4_^3−^	− 0.100	0.041	0.012	− 0.039	0.027	**0.886**
CO_3_^2−^	− 0.140	**− 0.692**	− 0.074	− 0.010	− 0.023	− 0.337
HCO_3_^−^	− 0.297	− 0.159	− 0.120	0.026	0.460	0.208
TH	**0.927**	0.160	0.147	0.188	0.053	− 0.033
Ca^2+^	**0.729**	− 0.069	− 0.382	− 0.182	− 0.231	0.092
Mg^2+^	**0.798**	0.215	0.326	0.313	0.085	− 0.050
K^+^	**0.807**	− 0.032	− 0.375	− 0.106	0.008	0.039
Na^+^	**0.931**	0.150	0.196	0.133	− 0.004	− 0.045
Surfactants	**0.732**	0.131	0.107	0.165	0.085	− 0.116
CO_2_	0.094	**0.845**	− 0.133	− 0.025	− 0.013	− 0.165
Phenol	0.196	0.078	− 0.015	− 0.077	**0.873**	− 0.086
Eigenvalue	7.984	1.960	1.458	1.307	1.160	1.015
% of variance	42.023	10.315	7.676	6.881	6.105	5.340
Cumulative %	42.023	52.337	60.013	66.894	73.000	78.339

#### PC 1

PC 1 explains 42.02% of the total variance (Table 2). It is characterized by high positive weight values TH, Ca^2+^, Mg^2+^, TDS, Na^+^, K^+^, Cl^−^, SO_4_^2−^ and surfactants. As Table 3 indicates, there is a strong positive correlation between TDS and Ca^2+^, Mg^2+^, Na^+^, K^+^, SO_4_^2−^, Cl^−^. These ions are the major contributors to the total dissolved solids. Additionally, these ions correlate with each other. These results show that the groundwater has suffered serious mineralization process from the natural condition of the salt pond (Allen and Suchy 2001). Moreover, since TDS correlates with surfactants and surfactants correlate with the above-mentioned ions, it is clear that there is a similarity across parameters.

**Table 3:** Pearson correlation matrix for 19 hydrochemical variables (whole dataset)*

	pH	TPH	TDS	Cl^−^	SO_4_^2−^	NH_4_^+^	NO_2_^−^	NO_3_^−^	PO_4_^3−^	CO_3_^2−^	HCO_3_^−^	TH	Ca^2+^	Mg^2+^	K^+^	Na^+^	Surfactants	CO_2_	Phenol index
pH	**1**	0.062	− 0.389	− 0.420	− 0.296		− 0.061	− 0.148		**0.578**	0.164	− 0.342	− 0.132	− 0.346	− 0.141	− 0.354	− 0.373	− **0.731**	− 0.072
TPH		**1**	0.132	0.106	0.117	0.344	− 0.070		− 0.128		− 0.165	0.142		0.140	0.175	0.123	0.188		
TDS			**1**	**0.971**	**0.941**	0.373	0.290	0.479	− 0.117	− 0.235	− 0.307	**0.972**	**0.509**	**0.942**	**0.607**	**0.979**	**0.715**	0.234	0.171
Cl^−^				**1**	**0.878**	0.380	0.324	0.475	− 0.119	− 0.251	− 0.339	**0.934**	**0.513**	**0.915**	**0.567**	**0.951**	**0.683**	0.287	0.096
SO_4_^2−^					**1**	0.307	0.404	**0.516**	− 0.106	− 0.198	− 0.282	**0.914**	**0.520**	**0.847**	**0.667**	**0.926**	**0.734**	0.130	0.222
NH_4_^+^						**1**	0.166	0.238		− 0.089		0.419	0.131	0.499	0.101	0.373	0.300	− 0.059	− 0.062
NO_2_^−^							**1**	0.464		− 0.069	− 0.157	0.256		0.304		0.335	0.276		− 0.070
NO_3_^−^								**1**		− 0.215	− 0.124	**0.504**	0.181	**0.504**	0.342	**0.512**	0.432		0.347
PO_4_^3−^									**1**	− 0.125	0.070	− 0.115	− 0.085	− 0.100	− 0.081	− 0.113	− 0.129		
CO_3_^2−^										**1**	0.124	− 0.238	− 0.098	− 0.237	− 0.138	− 0.208	− 0.075	− 0.353	− 0.091
HCO_3_^−^											**1**	− 0.307	− 0.186	− 0.271	− 0.193	− 0.310	− 0.100	− 0.112	0.063
TH												**1**	**0.521**	**0.945**	**0.628**	**0.961**	**0.706**	0.178	0.228
Ca^2+^													**1**	0.299	**0.713**	**0.558**	0.391	0.080	
Mg^2+^														**1**	0.409	**0.925**	**0.641**	0.199	0.213
K^+^															**1**	**0.592**	**0.532**	0.127	0.148
Na^+^																**1**	**0.705**	0.181	0.183
Surfactants																	**1**	0.184	0.126
CO_2_																		**1**	0.068
Phenol index																			**1**

*Insignificant coefficients at the 0.05 level are removed. Bold values represent significant coefficients higher than 0.5

There also is a clear correlation between TH and subsequent ions: Ca^2+^, Mg^2+^, Cl^−^, SO_4_^2−^ (Table 3). In addition, it can be seen that all these ions correlate with each other. This correlation points to the existence of non-carbonate, or constant hardness, (MeSO_4_, MeCl_2_, where Me—Ca, Mg), which is difficult to remove. It is clear from Table 3 that there is no correlation between carbonate ions and the hardness metals ions, which suggests a weak temporary hardness. This factor can be explained by the natural conditions of the site. In contrast, surfactants are synthetic compounds. Surfactants are produced for cleaning and washing operations (West and Harwell 1992). Their existence in groundwater is not natural.

#### PC 2

PC 2 explains 10.32% of the total variance (Table 2) with negative weight values of pH and CO_3_^2−^, and positive value of CO_2_. It is important to note a correlation between CO_2_, CO_3_^2−^ and pH (Table 3), which points to alkalinity reactions in the groundwater (Eq. 6). The relationship exists between these parameters and CO_2_, which potentially could be described a process of CO_2_ creation or the presence of the CO_2_ as an atmospheric gas in the unsaturated zone (Hem 1970). Moreover, the high concentration of chlorides in wastewater coupled with the natural salt water leads to changing pH in groundwater by decreasing pH. These processes are naturally based.

6 $${\text{Alk}} = 2\left[ {{\text{CO}}_{3}^{2 - } } \right] + \left[ {{\text{HCO}}_{3}^{ - } } \right] + \left[ {{\text{OH}}^{ - } } \right]{-}\left[ {{\text{H}}^{ + } } \right].$$

#### PC 3

Factor 3 is characterized by a positive value of nitrite ion (Table 2) and contributes 7.68% to the total variance. NO_2_^−^ does not correlate with any chemicals. The presence of the parameter could be explained as a semi-product of the natural denitrification/deammonification processes in the groundwater environment according to Hisckock et al. (1991).

#### PC 4

TPH and ammonia ion represent PC 4 and account for 6.89% of the total contamination (Table 2). Both chemicals have no correlation according Table 3, which shows their independence on the other variables. This level of petroleum hydrocarbons in drinking water can lead to damage of the nervous system and carcinogen and narcotic effects associated caused by some hydrocarbons (Logeshwaran et al. 2018). In addition, even a few micrograms of TPH per litre deteriorate the odour and taste of the contaminated water. The high loading of NH_4_^+^ is associated with extremally high concentrations of ammonia in discharges (Radelyuk et al. 2019). Hence, the amount of ammonia is not degraded during the saturation processes and some traces still presence in the groundwater. This factor is certainly attributed to groundwater pollution from the petrochemical industry.

#### PC 5

PC 5 is characterized by positive value of phenols (Table 2), which accounts for 6.11% of the whole contamination. This parameter is characterized as a very toxic pollutant. Concentrations of the phenolic compounds probably exceed the permissible level (Table 1); the exposure is evaluated as a potential risk for public health. The loading of this parameter is directly related to the specification of petrochemical wastewater.

#### PC 6

One more significant factor belongs to the influence of phosphate-ions and is rated by 5.34% of the total variance (Table 2). It should be pointed out that the enterprise does not provide monitoring of phosphate concentration in the discharges. Nevertheless, the refining process is associated with a vast number of washing processes, which leads to big consumption of different detergents, which contain phosphate substances. As the rocks and fertilizers are absent in the study area (Rao and Prasad 1997), we can conclude that the loading of the contaminant is an indicator of anthropogenic impact on the groundwater.

### Cluster analysis

**Fig. 9:**
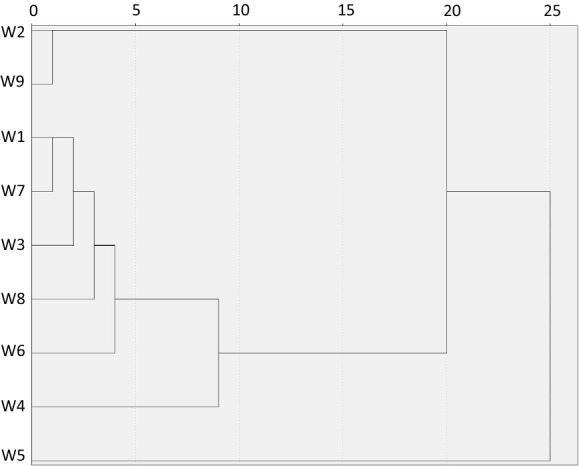
Dendrogram showing clustering of sampling sites according to groundwater characteristics (Ward Linkage. Euclidean Distance)

Based on the performed CA and results above, the study area was divided into three clusters. Figure 9 shows a dendrogram of all nine sampling sites into three statistically meaningful clusters yielded by cluster analysis. Cluster 1 combines observed wells W9 and W2. These wells are labelled as highly contaminated with the highest exceeding of many chemical parameters. Figure 4a shows their similarities in the distribution of pH, which is followed by host geology. The wells are situated on the southwest site from the pond and probably approve an assumption about direction of groundwater flow. Cluster 2 is formed by wells W7, W8, W1 and W3. These wells are located on the south and west sides of the pond and characterized by twofold characteristics: firstly, significant pollution rate, including the same concentrations of the TDS and TDS related chemicals and secondly, the equal temporal distribution of pH. It means that groundwater on that site is affected by pollutant transport from the pond in the same manner. Finally, Cluster 3 is represented by wells W6, W4 and W5. All wells are located north of the pond and are characterized by lower concentrations of the pollutants compared to other wells. We may consider that groundwater flow originates from east to west, and potential hazard exists for rural inhabitants towards to west and south-west direction from the pond.

### The Heckman selection model

This study uses the Heckman selection model to estimate relationships between total contamination and other characteristics, especially, significance of toxicity rate. If we adapt Eqs. (4) and (5) for our case according Stata manual (STATA 2013), we can represent the equations respectively as:

7 $$\% {\text{ of exceeding }} = \beta_{1} {\text{chemical }} + \beta_{2} {\text{concentration }} + u_{i} ,$$

and we assumed that “% of exceeding” is estimated if

8 $$\gamma_{1} {\text{toxicity }} + \gamma_{2} {\text{number}}\;{\text{of}}\;{\text{well}} + \gamma_{3} {\text{chemical}}\gamma_{4} + {\text{concentration }} + \nu_{i} > \, 0,$$

where $ui$ and $\nu i$ have positive correlation ρ.

Table 4 shows the selected variables used in this analysis and their descriptive statistics. The first dependent variable (*D*_*i*_) represents toxicity of the chosen chemical. The value equals 1 if the pollutant is toxic and 0 if not. The second set of dependent variables (*Y*_*i*_) includes percentage of exceeding. This characteristic mathematically represents rate of contamination. Mean percentage of selected (toxic or non-toxic) exceeding was calculated. For example, if the concentration of TPH measurement was 0.25 mg/L, but standard value is no more than 0.1 mg/L, then dependent variable equals 250%. This variable includes only exceeded values. Otherwise, if the value is normal, a cell in a matrix is empty. Numbers in parentheses are standard deviations of the average values. Set of control variables (*X*_*i*_) includes chosen contaminants, their concentrations and locations. According requirements (Kazakhstan 2015), TH, TPH, and Na^+^ are considered as hazardous for public health and rated with value 1.0 for the variable *D*_*i*_. TDS, sulphates and chlorides are considered as non-toxic and were rated as value 0.0 for the variable *D*_*i*_. We encrypted TDS, Cl^−^, SO_4_^2−^, Na^+^, TH and TPH in the table of variables as “1”, “2”, “3”, “4”, “5” and “6”, respectively. The contaminants are not a subject for assessment in this analysis.

**Table 4:** Selected variables characteristics*

Variable	Toxic	Non-toxic
Number of observations	324	351
	48.0%	52.0%
Number (%) of exceeded values	255 (78.7)	248 (70.7)
Dependent variables		
Toxic contaminant	1.0	0.0
% of exceeding	664 (1042)	862 (1526)

*Statistics of chosen chemicals is available from Table 1

Table 5 presents the estimation for this type of analysis. Rho has a positive value, which means that it is possible to estimate relationships between chosen variables and final contamination. All variables, excluding number of well (which represents location of the wells), are considered as significant. The concentration of pollutants has the greatest influence on total contamination. Positive value explains likelihood of potential hazard for people health. Obviously, the high concentrations of the pollutants lead to deterioration of health, especially during long-term exposure. In our case, 503 of 675 values exceed acceptable limits by 7–8 times averagely. The variable of toxicity rate is the second significant factor. This variable reflects to lower percentage of exceedings for toxic contaminants than for non-toxic, instead of higher number of exceeded values for toxic contaminants than non-toxic. Our hypothesize assumes that even if the concentration of the toxic contaminant exceeds the standard by just a few units, the toxic properties could be much more dangerous for human health, compared with the consumption of highly polluted water by non-toxic contaminants. The independent chemicals represent the third significant variable. Individual characteristics of chosen chemicals are explained in sub-section “Groundwater quality parameters”. The location of the well is rated as not significant parameter. Nevertheless, the investigation of hydrogeological characteristics deserves attention in the future work and determines the spread of contamination.

**Table 5:** Estimated results of the Heckman selection model (two-step) for selected chemicals

Variable	Coefficient	Std. Err	Z-statistic
Chemical	− 0.156	0.074	− 2.11
Concentration	1.576	0.260	6.07
Toxicity rate	0.789	0.245	3.22
Number of well	0.020	0.025	0.83
Rho	1.0		

## Conclusions

This study investigated the current situation of groundwater safety for public health surrounding a contaminated site in Kazakhstan. The results show that PCAs have high loading of anthropogenic contamination to groundwater from the oil refinery industry coupled with natural geochemical processes. In addition, exceeding concentrations of hazardous substances, including TPH, phenols, TH, and TDS were identified. By means of cluster analysis we were able to combine the examined wells in three groups according to the concentrations of chemicals and their locations. Highly polluted groundwater was distributed especially in west and south-west direction from the pond. The results enable the prediction of the groundwater flow in the study area as well as the estimation of sites heavily affected by contamination. The usage of Heckman selection model, to the authors’ knowledge, is the first attempt in the literature, applied to evaluation of environmental factors. According to obtained data from Heckman analysis, focus should be paid to the distribution of toxic contaminants.

For this purpose, further research considers: (1) Groundwater modelling for definite identification of groundwater flow and potentially affected rural areas; (2) Contamination transport modelling, as the industry continue polluting the environment, the assessment of present and future hazards is highly needed; (3) Development of a remediation plan, which has to be built on the qualitative studies (1) and (2).

This study might be used as a trigger to drive and engage all stakeholders into the transparent dialogue about potential consequences of non-sustainable wastewater management at oil refinery industry. The potential actions might include implementation of successful legislative standards, development of new efficient monitoring programs, stimulation the industry to innovative and water-saving treatment methods and a creation of a site contamination/remediation programs.

This research has several limitations. Firstly, the limited dataset covers only period from 2013 to 2019. Secondly, despite of the concentrations of TPH are identified, the lack of data on specific hydrocarbon type such as PAH and BTEX limited the analysis on the toxicity. Thirdly, the lack of access to hydrogeological data limited the accuracy of the ground water flow estimation. Authors of this paper recommend initiating a dialogue between industry, government, and academia for research-based decision-making in this area.

## Abbreviations

Alk: Alkalinity
BTEX: Benzene, toluene, ethylbenzene and xylenes
CA: Cluster analysis
EPA: United States Environmental Protection Agency
EU: European Union
GW: Groundwater
km: Kilometers
KZ: Kazakhstan
m: Meters
PAH: Polycyclic aromatic hydrocarbons
PC: Principal component
PCA: Principal component analysis
TDS: Total dissolved solids
TH: Total hardness
TPH: Total petroleum hydrocarbons
UN: United Nations
WHO: World Health Organization
